# Fourier Transform Infrared Spectroscopy Analysis as a Tool to Address Aβ Impact on Extracellular Vesicles

**DOI:** 10.3390/molecules30020258

**Published:** 2025-01-10

**Authors:** Margarida Vaz, Tânia Soares Martins, Kevin Leandro, Luís Pereira de Almeida, Odete A. B. da Cruz e Silva, Alexandra Nunes, Ana Gabriela Henriques

**Affiliations:** 1 Neuroscience and Signalling Group, Institute of Biomedicine (iBiMED), Department of Medical Sciences, University of Aveiro, 3810-193 Aveiro, Portugal; margaridavaz@ua.pt (M.V.); martinstania@ua.pt (T.S.M.); odetecs@ua.pt (O.A.B.d.C.e.S.); 2 Center for Neuroscience and Cell Biology, Faculty of Pharmacy, University of Coimbra, 3004-504 Coimbra, Portugal; kleandro@cnc.uc.pt (K.L.); luispa@cnc.uc.pt (L.P.d.A.); 3 ViraVector–Viral Vector for Gene Transfer Core Facility, University of Coimbra, 3004-504 Coimbra, Portugal; 4 Institute of Biomedicine (iBiMED), Department of Medical Sciences, University of Aveiro, 3810-193 Aveiro, Portugal; alexandranunes@ua.pt

**Keywords:** FTIR, Aβ, Alzheimer’s disease, extracellular vesicles, Neuro-2a cells

## Abstract

Alzheimer’s disease is a challenge in modern healthcare due to its complex etiology and increasing prevalence. Despite advances, further understanding of Alzheimer’s disease pathophysiology is needed, particularly the role of Aβ neurotoxic peptide. Fourier transform infrared spectroscopy (FTIR) has shown potential as a screening tool for several pathologies, including Alzheimer’s disease. Nonetheless, limited research has explored Aβ direct effects on neurons and extracellular vesicles metabolic profiles. Hence, this study aims to investigate Aβ impact on the spectroscopic profiles of neuronal-like cells (N2a) and N2a-derived extracellular vesicles, employing FTIR spectroscopy and focusing on the 1280–900 cm^−1^ region. A comprehensive analysis of spectral data was carried out, including multivariate partial least squares (PLS) analysis and peak intensities analysis. PLS analysis revealed moderate to strong correlations within this spectral region for both N2a and N2a-derived extracellular vesicles. The peak intensity analysis revealed additional peaks with significant differences in EVs’ spectra relative to N2a, following Aβ treatment. Specifically, Aβ seems to cause alterations in protein phosphorylation and in the nucleic acids content of extracellular vesicles. These findings support that Aβ’s role in Alzheimer’s disease pathology may be mediated by extracellular vesicles and highlight FTIR’s potential for advancing Alzheimer’s disease research and clinical applications.

## 1. Introduction

Dementia affects more than 55 million people worldwide, a number expected to triple by 2050. Alzheimer’s disease (AD) is the main cause of dementia, contributing to 60–70% of cases [1]. This progressive neurodegenerative disorder is clinically characterized by cognitive decline, affecting memory and orientation, having a huge impact on the patient’s quality of life. At the histopathological level, the two main AD hallmarks are amyloid-beta peptides (Aβ) deposition into extracellular senile plaques (SPs) and the intracellular formation of neurofibrillary tangles, due to hyperphosphorylated tau protein aggregation [2].

Aβ is a small peptide generated from amyloid precursor protein (APP) proteolytic cleavage. The latter is a transmembrane protein highly expressed in neurons. Amyloidogenic APP processing comprises an initial proteolytic cleavage by β-secretase leading to the release of soluble fragments, the amyloid precursor proteins (sAPP β), and a C-terminal peptide that is further cleaved by γ-secretase, generating the amyloid peptides [3]. Aβ peptides can vary in length, with the two major species present in the brain, Aβ_1–40_ and Aβ_1–42_, being the last more prone to aggregation and the primary component of SPs [2]. Once generated, the Aβ monomers tend to assemble first into Aβ oligomers (AβOs), then form protofibrils, that ultimately elongate into insoluble fibrils and accumulate into SPs [4]. Although SPs are one of AD’s hallmarks, AβOs are a major neurotoxic species. Soluble AβOs are highly toxic and can cause synaptic dysfunction by interacting with cell-surface neuronal receptors, impairing long-term potentiation [5]. AβOs, as well as Aβ protofibrils and fibrils, can activate microglia, leading to the release of pro-inflammatory cytokines, and inducing neuroinflammation, a key event in AD pathogenesis [3]. Additionally, AβOs can impair mitochondria function, disrupt intracellular calcium homeostasis, and induce reactive oxygen species production.

A large body of evidence places Aβ accumulation and aggregation at the center of AD pathophysiology, but many other molecular alterations have been linked to the disease, including metabolic alterations. Distinct metabolic analysis has been carried out, including resorting to Fourier transform infrared (FTIR) spectroscopy. This technique is rapid and easy to perform, affordable, label-free, and requires very small sample volumes. It measures the vibrational energy of chemical bonds based on the infrared light absorbed by molecular bonds, providing valuable information simultaneously regarding samples’ main molecular contents: proteins, nucleic acids, lipids, and carbohydrates [6,7]. Although FTIR offers many advantages, other techniques such as Raman spectroscopy and nuclear magnetic resonance (NMR) are also widely used in the analysis of biomolecules. FTIR and Raman aim to identify functional groups in samples whereas NMR is applied to the identification of specific molecules, making these techniques complementary. While FTIR measures the frequencies at which samples absorb radiation and is sensitive to hetero-nuclear functional groups and polar bonds, Raman relies on inelastic light scattering to analyze molecular vibrations and is sensitive to nonpolar and homo-nuclear bonds, allowing the analysis of samples in aqueous environments. However, Raman-generated signals are usually weak and it can be necessary to apply signal enhancement techniques or denoising algorithms to improve signal [8,9]. NMR spectroscopy offers high-resolution structural data, which is relevant to identifying the exact structure of a molecule, being more specific to the study of metabolites than FTIR or Raman spectroscopic techniques [10,11]. FTIR spectroscopy is a powerful analytical tool for characterizing metabolic profiles, highly reproducible and it has been applied successfully to discriminate control from AD cases using biofluids [6,7]. Moreover, FTIR-based metabolic analysis has been used to understand AD pathology, focusing on the study of Aβ structure and aggregation [12,13,14,15,16], including the evaluation of how aluminum [17] or oxidative stress [18,19] impact this process.

Extracellular vesicles (EVs) are nanovesicles, ranging from 30 to 150 nm, secreted by all cell types and recognized as important mediators in cell-to-cell communication. These vesicles have an endocytic origin and their cargo comprises proteins, nucleic acids, and lipids [20]. In AD, it has been reported that EVs can carry Aβ, along with other proteins important in disease pathogenesis, such as tau, further supporting EVs’ involvement in disease pathogenesis [21].

Metabolomics has been applied to EVs derived from serum samples of AD cases [7,22] and brain tissue [23], but there is a need to further explore the alterations in EVs’ metabolic profile relevant to AD. Neuronal cultures mimicking AD conditions represent an attractive model to address EVs’ role in disease pathology. Since Aβ is a key player in AD, the aim of this work was to analyze the impact of Aβ on the spectroscopic profile of a neuronal-like cell line (N2a) and the respective derived EVs, by FTIR analysis, specifically focusing on the 1280–900 cm^−1^ region. This will shed light on Aβ-induced metabolic alterations, and their ability to be mediated by EVs, in the context of AD pathology.

## 2. Results and Discussion

### 2.1. EVs’ Isolation and Characterization

**Figure 1:**
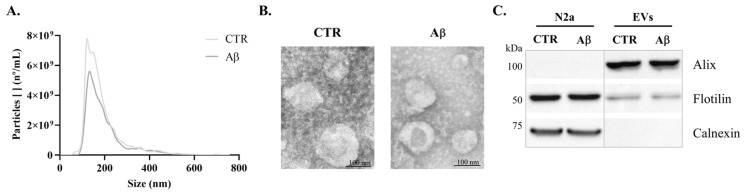
**Characterization of N2a-derived EVs.** EVs were isolated from the control and Aβ-treated cells. (**A**) Size distribution curves of EVs obtained by NTA. (**B**) Representative TEM image of the isolated EVs. (**C**) EVs markers (Alix and Flotilin) and negative marker (Calnexin) detected by WB.

Prior to the utilization of FTIR analysis, EVs were isolated by ultracentrifugation from conditioned media of N2a, which were treated with or without Aβ. EVs were further characterized by transmission electron microscopy (TEM), nanoparticle tracking analysis (NTA), and Western blot (WB), to evaluate the nature of EVs’ preparation. The size distribution curve obtained by NTA revealed that the isolated particles were within the expected exosome size range (Figure 1A), for both the control and Aβ-treated conditions (mode size of 121 nm and 129 nm, respectively). A tendency towards a decreased number of EVs upon Aβ addition was observed. The TEM analysis confirmed that isolated EVs exhibit the expected morphology and size (Figure 1B). Additionally, the presence of the EVs markers, Alix and Flotilin, and the absence of the negative cellular marker Calnexin, detected by WB, supported the conclusion that the preparations were indeed enriched in EVs (Figure 1C).

### 2.2. FTIR Spectra Overview Following Aβ Treatment

**Figure 2:**
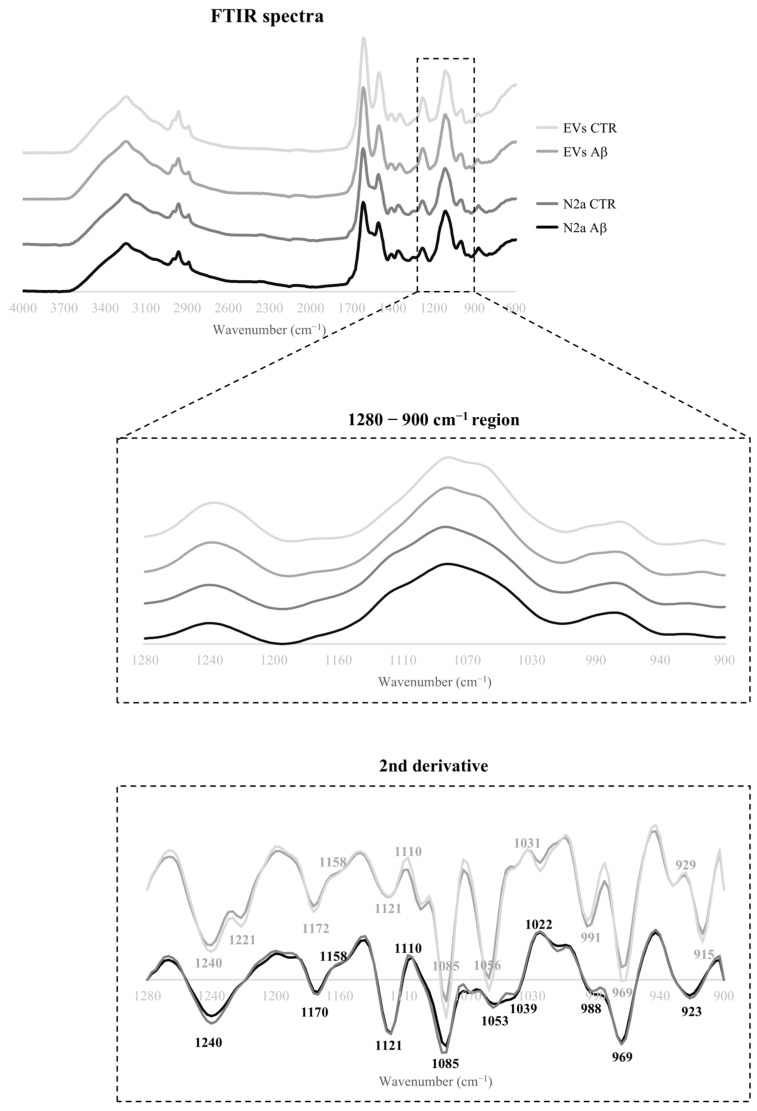
**N2a and N2a-derived EVs FTIR spectra.** Average pre-processed FTIR spectra of the 4000–600 cm^−1^ region from N2a and N2a-derived EVs. The FTIR spectra of the 1280–900 cm^−1^ region and the respective second derivative are highlighted.

The Aβ induced effects on the spectroscopic profile of N2a and N2a-derived EVs were evaluated by FTIR analysis. The 4000–600 cm^−1^ spectral region of both cells and EVs was baseline corrected, and area normalized (Figure 2). Overall, the spectra profiles of N2a and N2a-derived EVs were similar, even after Aβ treatment.

In the spectral region between 3000 and 2800 cm^−1^, signals related to CH_3_ asymmetric and symmetric stretching could be found, reflecting mainly the lipids composition [24], with no obvious differences between the control and Aβ-treated groups.

The spectral region associated with proteins is in the range of 1700–1500 cm^−1^, comprising two main bands: the amide I at 1640 cm^−1^ assigned to C=O and C–N stretching; and the amide II at 1535 cm^−1^ assigned to C–N stretching and C–N–H bending [24]. An additional small band can be found in this region, between 1760 and 1730 cm^−1^ that is assigned to C=O stretching of the lipid molecules within fatty acids and glycerol, that can also be used to measure lipid peroxidation [25]. For this region, both peaks are, in general, more intense in the N2a-derived EVs than in the N2a lysates.

The region between 1280 and 900 cm^−1^ of the spectra contains bands mainly associated with carbohydrates and nucleic acids, although few assignments related to protein phosphorylation and phospholipids content can also be proposed for some peaks in this region [24,26]. Differences could be observed between EVs and N2a in the spectra absorbance profile for this region.

Furthermore, partial least squares (PLS) analysis showed no clear discrimination between the control and Aβ groups for the 3000–2800 cm^−1^ (assigned to lipids) and 1700–1500 cm^−1^ (associated with proteins) spectral regions. Therefore, since discrimination was obtained for the 1280–900 cm^−1^ region and previous reports showed that this region holds a discrimination value between serum-derived EVs from the control and AD cases [7], this region was selected for further analysis. After applying the second derivative to the 1280–900 cm^−1^ region, several peaks were identified, and the respective proposed assignments are presented in Table 1.

### 2.3. Multivariate Analysis of the 1280–900 cm^−1^ Region

**Figure 3:**
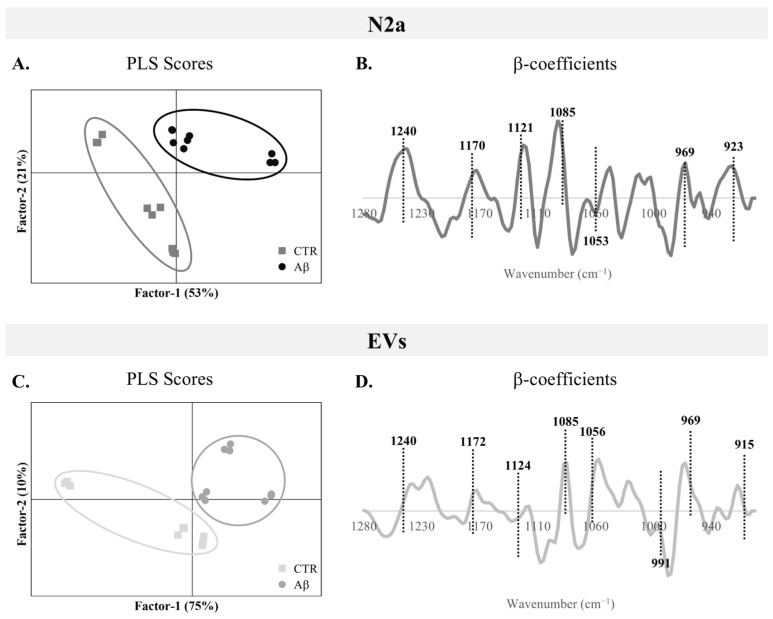
**PLS analysis of the 1280–900 cm^−1^ region of FTIR spectra.** (**A**) PLS scores plots and (**B**) respective β-coefficients plots of factor 1 for N2a. (**C**) PLS scores plots and (**D**) respective β-coefficients plots of factor 1 for N2a-derived EVs.

To assess the overall alterations in the 1280–900 cm^−1^ spectroscopic region of N2a and N2a-derived EVs upon Aβ treatment, a PLS analysis was performed. In score plots of the PLS analysis for N2a cells (Figure 3A) and N2a-derived EVs (Figure 3C), it is possible to observe good discrimination between the groups, since the Aβ-treated samples are located at positive factor-1 and most control samples are negative factor-1. This clear discrimination between the groups supports the metabolic alterations induced by Aβ in this AD-mimicking model, not only observed in N2a but also in the EVs secreted by these cells.

Indeed, both N2a and N2a-derived EVs presented moderate to strong correlation values of calibration (0.667 and 0.670, respectively), and similar cross-validation coefficients (Table 2). This is in agreement with previous work applying FTIR to AD cases and controls, where a moderate to strong correlation was observed for serum-derived EVs in the carbohydrates and nucleic acids FTIR spectral region [7].

β-Coefficient plots allow identification of the peaks responsible for the discrimination and that are more related to each condition clusters. Both Aβ-treated groups of N2a and N2a-derived EVs (Figure 3B and Figure 3D, respectively) are characterized by the peaks located at 1240 cm^−1^ (${\mathrm{PO}}_{2}^{-}$ asymmetric stretching of phosphorylated proteins, phospholipids and nucleic acids; C–O–P stretching (phosphorylated proteins and lipids)), 1170/1172 cm^−1^ (C–O stretching of proteins, carbohydrates and nucleic acids; CO–O–C asymmetric stretching of ester bonds in cholesterol esters of carbohydrates), 1085 cm^−1^ (${\mathrm{PO}}_{2}^{-}$ symmetric stretching of phosphorylated proteins, phospholipids, glycogen, and nucleic acids; C–O–P stretching of phosphorylated proteins and lipids), 969 cm^−1^ (${\mathrm{PO}}_{3}^{2-}$ symmetric stretching of nucleic acids and phosphorylated proteins; C–C, C–O and C–N–C stretching of phosphodiester, DNA and RNA), 923 cm^−1^ (left-handed helix of DNA), and 915 cm^−1^ (ribose ring vibrations of nucleic acids). Furthermore, the peak at 1121/1124 cm^−1^ (C–O stretching of carbohydrates and ribose in RNA) was associated with Aβ-treated N2a and the peak 1053/1056 cm^−1^ (C–O stretching and C–OH bending of nucleic acids, phospholipids and carbohydrates) characterized EVs from Aβ-treated cells. Interestingly, an additional peak at 991 cm^−1^ (C–O stretching from RNA and carbohydrates; C–C stretching of DNA backbone) was associated with the control EVs.

Therefore, Aβ treatment altered the spectroscopic profile of N2a and N2a-derived EVs, evident in the 1280–900 cm^−1^ region, causing alterations to the peaks that are mostly assigned to nucleic acids content, carbohydrates, but also phospholipids and phosphorylated proteins.

### 2.4. Peak Intensity Analysis

**Figure 4:**
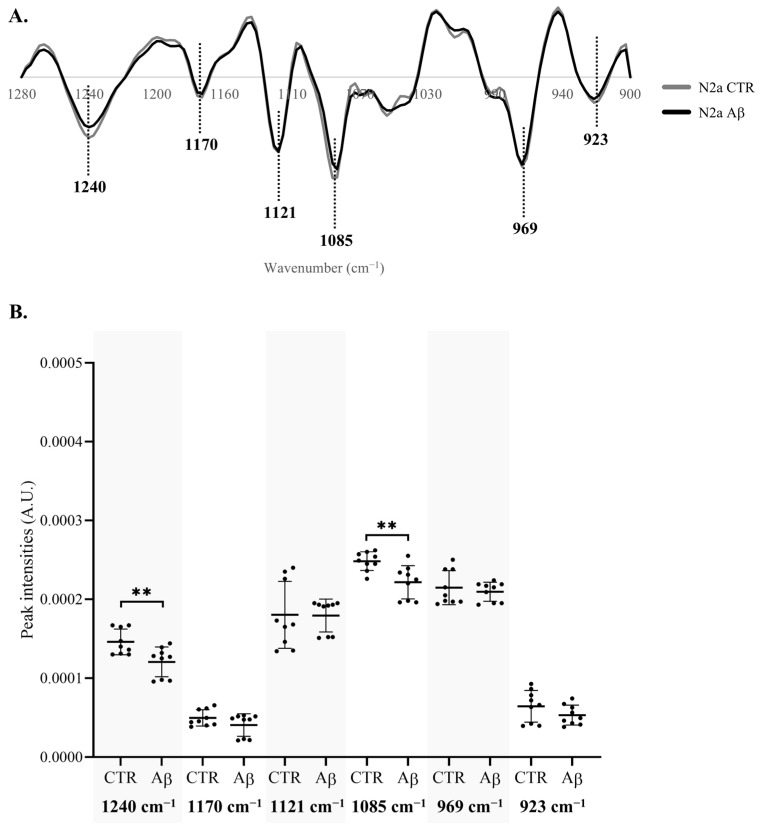
**Analysis of peak intensity in the 1280–900 cm^−1^ region of N2a FTIR spectra.** (**A**) Mean second derivative spectra of N2a. (**B**) Peak intensity analysis of N2a spectra. Abbreviations: A.U., arbitrary units. ** *p* < 0.01.

**Figure 5:**
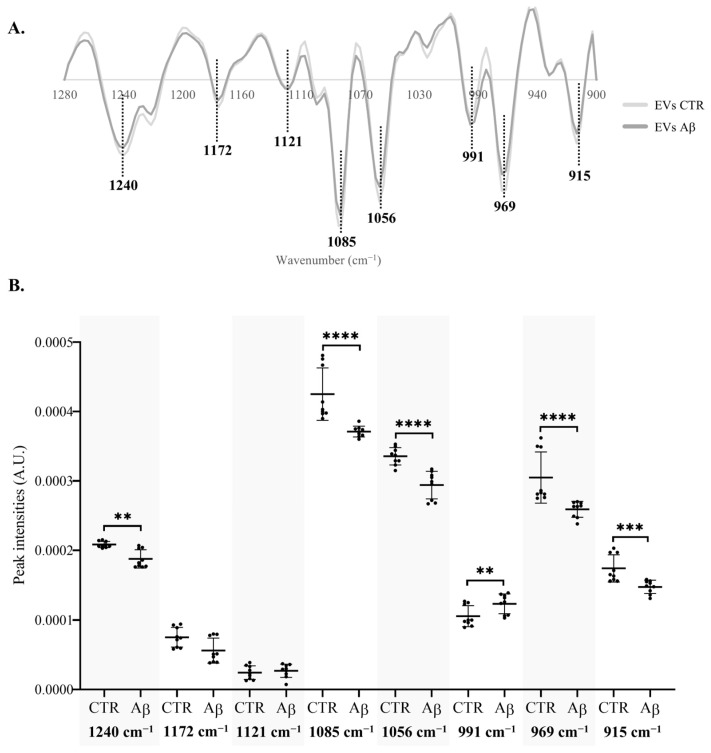
**Analysis of peak intensity in the 1280–900 cm^−1^ region of N2a-derived EVs.** (**A**) Mean second derivative spectra of N2a-derived EVs. (**B**) Peak intensity analysis of N2a-derived EVs. Abbreviations: A.U., arbitrary units. ** *p* < 0.01,*** *p* < 0.001; **** *p* < 0.0001.

To better understand the specific spectroscopic alterations caused by Aβ, in the 1280–900 cm^−1^ region of N2a (Figure 4A) and N2a-derived EVs (Figure 5A), the intensity of the peaks resulting from the second derivative spectra was quantified considering those also identified in the β-coefficient plot of PLS analysis. The intensity of the peak at 1053 cm^−1^ in N2a second derivative spectra was not quantified due to a peak shift between the samples of the same condition.

Significant decreases were found in the intensity of peaks 1240 cm^−1^ and 1085 cm^−1^ of N2a after Aβ treatment (Figure 4B), which were assigned to the asymmetric and symmetric stretching of ${\mathrm{PO}}_{2}^{-}$ (respectively) of phosphorylated proteins, phospholipids and nucleic acids and C–O–P stretching of phosphorylated proteins and lipids. The decrease in these peaks’ intensities was also evident in N2a-derived EVs (Figure 5B). Indeed, the peak at 1085 cm^−1^ presented a greater decrease in N2a-derived EVs than in N2a. Since these peaks may reflect the phosphorylated proteins’ content [30,31,32], these results can suggest that Aβ can cause alterations in protein phosphorylation, a key event in AD pathogenesis. This is also consistent with the reported effects of Aβ peptide on the activity of kinases and phosphatases [33], potentially impacting the phosphorylation states of several proteins including tau [34]. Of relevance, phosphorylated tau species have been reportedly altered in blood-derived EVs of AD cases [35,36,37,38]. On the other hand, these peaks can also represent alterations in the DNA/RNA phosphate backbone, indicating that Aβ treatment may impact the nucleic acid content.

For EVs, it was possible to observe significant differences in four additional peaks than in N2a cells (Figure 5B). Three peaks, 1056 cm^−1^, 969 cm^−1^, and 915 cm^−1^ were significantly decreased. These are mainly assigned to molecular vibrations of C–O, C–C and ${\mathrm{PO}}_{2}^{-}$ of nucleic acids, phospholipids, phosphorylated proteins, and carbohydrates [39]. Interestingly, a significant increase was observed in the intensity of peak 991 cm^−1^ that is mostly related to the stretching motions of nucleic acid ribose [39,40] but also of the DNA backbone. These alterations to the RNA/DNA content of EVs might be explained by the degradation of nucleic acids as a consequence, e.g., of oxidative stress, that is observed in AD [41]. The results also suggest that Aβ may impact neurons by altering the RNA and DNA content of EVs, potentially causing several alterations in recipient cells [42]. Indeed, EVs contain RNA, such as miRNA and mRNA, which can be exchanged between cells, impacting their function [43,44]. In AD, several miRNAs and mRNAs were shown to be altered in EVs derived from patients’ biofluids [21,45]. Additionally, an enrichment of mRNA related to inflammation and a depletion of synaptic signaling mRNAs in brain-derived EVs of AD patients was observed [46]. Therefore, during AD progression, cells can pack EVs with different RNA species, potentially modulating distinct pathways. However, it is not clear if this modulation can contribute to the disease progression or in opposition be beneficial since EVs were reported to have a dual role in AD [47].

Taken together, the data presented reveal that Aβ had a bigger impact on N2a-derived EVs FTIR profile than on N2a, causing mainly a decrease in the intensity of peaks associated with phosphate groups present at nucleic acids, carbohydrates, phosphorylated proteins, and phospholipids. This is consistent with previously published work that used human samples, in which FTIR analysis of this spectral region revealed a better discrimination in serum-derived EVs than in serum [7], between the AD and control cases. In both studies, a significant decrease was also observed in peaks assigned to RNA/DNA in human samples, although distinct peaks were found in cells- and serum-derived EVs due to the different nature of the samples. This ultimately supports that Aβ treatment is a promising in vitro model to study AD-related metabolic alterations.

## 3. Materials and Methods

### 3.1. Cell Culture and Aβ Treatment

Neuro-2a mouse neuroblastoma cells (N2a) were maintained in Dulbecco’s Modified Eagle Medium (DMEM) (52100 Gibco, Thermo Fisher Scientific, Waltham, MA, USA) supplemented with 10% fetal bovine serum (FBS) (10270 Gibco, Thermo Fisher Scientific, Waltham, MA, USA) and 1% of penicillin/streptomycin mix (L0022 Biowest, Nuaillé, France). Cells were cultured at 37 °C, in an incubator with 5% CO_2_ and 95% humidity. For Aβ experiments, N2a cells were seeded at a density of 1 × 10^5^ cells/cm^2^ in 6-well plates and treated with 10 µM of Aβ_25–35_ (A-2535 Genicbio, Shanghai, China), as previously described [48], in FBS free medium, for 48 h. Cell viability was assessed using resazurin assay (Figure S1). No significant effects on cell viability were observed under these Aβ treatment conditions. Three independent biological experiments were carried out.

### 3.2. EVs Isolation

Cells were treated as described and cell culture medium from ten 100 mm plates (density 1 × 10^5^ cells/cm^2^) per condition was collected for EVs isolation by ultracentrifugation. The conditioned medium was submitted to sequential low speed centrifugations of 300× *g* for 10 min, 2000× *g* for 10 min and 10,000× *g* for 30 min at 4 °C. Subsequentially, it was passed through a 0.2 µm filter and the supernatant was then ultracentrifuged at 100,000× *g* at 4 °C for 2 h, using an Ultracentrifuge (Optima L-80K—Beckman Coulter, Brea, CA, USA) to isolate EVs. The resulting pellet was washed in PBS and ultracentrifuged again at 100,000× *g* at 4 °C for 2 h. The final pellet containing EVs was resuspended in PBS.

### 3.3. Nanoparticle Tracking Analysis

EVs preparations were submitted to Nanoparticle tracking analysis (NTA) to determine EVs concentration and size distribution curves, using Nanosight NS300 (Malvern Instruments, Malvern, UK). Samples were diluted to achieve ideal particle per frame value of 20–100 particles/frame. The analysis was performed in duplicate for each sample. For each measurement, five videos were captured at room temperature (RT) with a syringe speed of 40 μL/s. Video analysis was performed with NTA software version 3.2 using a detection threshold of 5. Particle concentration was corrected by the dilution factor to determine the final concentration.

### 3.4. Transmission Electron Microscopy

For transmission electron microscopy (TEM) analysis, EVs preparations randomly selected were fixed with 2% paraformaldehyde and left to adsorb in 75 mesh Formvar/carbon grids. A 3% phosphotungstic acid solution was added, as a negative staining. TEM images were obtained using a Hitachi H-2700 transmission electron microscope (Hitachi, Tokyo, Japan) at 100 kV and images were captured using a slow-scan CCD camera.

### 3.5. Western Blot Analysis

Prior to immunodetection of EVs markers by Western blot (WB), the EVs preparations in PBS were lysed by mixing with an equal volume of RIPA buffer, with phosphatase and protease inhibitors. Protein concentration was determined by BCA protein assay and 50 µg of total protein was separated using a 5% to 20% gradient SDS-PAGE gel and electrophoretically transferred onto a nitrocellulose membrane. The nitrocellulose membranes were blocked in 5% non-fat dry milk and further incubated with the primary antibodies: anti-Alix (1:500) (sc-53538), anti-Flotillin (1:500) (sc-74566) (Santa Cruz Biotechnology, Dallas, TX, USA), and anti-Calnexin (1:1000) (ADI-SPA-860) (Enzo, Farmingdale, NY, USA). Then, the membranes were incubated with the HRP-conjugated secondary antibodies anti-mouse (7076S) or anti-rabbit (7074S) (Cell Signaling Technology, Danvers, MA, USA). Protein bands were detected using the chemiluminescence reagent ECL Select (GE Healthcare Life Sciences, Milwaukee, WI, USA) and images obtained with the ChemiDoc™ gel imaging system (Bio-Rad, Hercules, CA, USA).

### 3.6. FTIR Samples Preparation and Spectra Acquisition

For the preparation of N2a samples for FTIR analysis, following treatments, the cells were counted in a hemocytometer, using Trypan Blue, and aliquots with 1 × 10^5^ cells were centrifuged at 300 × *g* for 3 min at RT. The cells pellets were frozen at −30 °C until FTIR analysis.

FTIR spectra were acquired using an Attenuated Total Reflectance (ATR)-FTIR Bruker Alpha Platinum spectrometer (Bruker, Billerica, MA, USA), coupled to OPUS software (V.7, Bruker, Billerica, MA, USA). Cell pellets were resuspended in PBS and 5 µL of cells or EVs suspension were placed at the center of the ATR diamond and air dried. Three independent biological experiments were carried out and, for each sample, three technical replicates were acquired. Between the samples, the ATR crystal was cleaned with 70% ethanol and distilled water, and a background spectrum was acquired against air. Spectra were acquired in the region of 4000–600 cm^−1^, with a resolution of 8 cm^−1^ and 64 co-added scans. RT and humidity were maintained constant, during acquisition, at 23 °C and 35%, respectively.

### 3.7. FTIR Spectra Pre-Processing and Multivariate Analysis

FTIR spectra were exported in OPUS format and analyzed using the Unscrambler X software (V.10.4., Camo Analytics, Oslo, Norway).

From the initial spectra, the 1280–900 cm^−1^ region was baseline corrected, and the area normalized. Further, the second derivative with Savitzky–Golay algorithm with 3 smoothing points was applied so that the spectra bands would be deconvoluted to obtain more detailed information. After pre-processing, the spectra were subjected to multivariate supervised approach partial least squares (PLS) analysis.

### 3.8. FTIR Spectra Peak Intensities Analysis

To calculate the intensity of the spectral bands, the second derivative spectra was inverted by factoring by −1 [49]. Then, the intensity of peaks assigned to 1240 cm^−1^, 1170 cm^−1^, 1121 cm^−1^, 1085 cm^−1^, 969 cm^−1^, and 903 cm^−1^ for cells samples and 1240 cm^−1^, 1172 cm^−1^, 1121 cm^−1^, 1102 cm^−1^, 1085 cm^−1^, 1056 cm^−1^, 991 cm^−1^, 969 cm^−1^, and 915 cm^−1^ for EVs samples were extracted.

To compare peak intensities, data distribution was assessed, and non-parametric Mann–Whitney test was applied using GraphPad Prism 8.0.2 software (GraphPad Software, San Diego, CA, USA). The results were expressed as mean ± standard deviation and were considered statistically significant when *p* < 0.05.

## 4. Conclusions

In summary, the presented FTIR spectroscopy study allowed to investigate the influence of Aβ treatment on the spectroscopic profiles of a neuronal cell model and the released EVs. Aβ treatment leads to alterations in the 1280–900 cm^−1^ region of FTIR spectra, exhibiting a higher number of significantly altered peaks in EVs than in cells, which were mainly assigned to nucleic acids/carbohydrates. Hence, Aβ effects in AD can be mediated by alterations in EVs content. Exploring EVs cargo may uncover novel disease profiles or targets, potentially contributing to advancements in AD diagnostics and/or therapeutics.

## Figures and Tables

**Table 1:** Proposed peak assignments for N2a cells and N2a-derived EVs for the 1280–900 cm^−1^ region of FTIR spectra.

Peak (cm^−1^)	Proposed Assignments	Ref
1240 1221 *	${\mathrm{PO}}_{2}^{-}$ asymmetric stretching of phosphorylated proteins, phospholipids, and nucleic acids C–O–P stretching (phosphorylated proteins and lipids)	[24,27,28]
1172 * 1170 #	C–O stretching of C–OH groups of serine, threonine, and tyrosine in proteins and of carbohydrates (proteins, carbohydrates/glycogen) C–O stretching (nucleic acids) CO–O–C asymmetric stretching of ester bonds in cholesteryl esters (carbohydrates)	[26,27]
1158	C–O stretching, C–C stretching, and C–O–H bending (glycogen/carbohydrate and proteins)	[26,27,28]
1121	C–O stretching of C–OH group of ribose and carbohydrates (RNA, carbohydrates)	[26,27]
1110	CC–OC stretching of ribose (RNA) Symmetric stretching C–O–P Carbohydrates	[26,27]
1085	${\mathrm{PO}}_{2}^{-}$ symmetric stretching of phosphorylated proteins, phospholipids, glycogen, and nucleic acids C–O–P stretching (phosphorylated proteins and lipids)	[24,26,27,28]
1056 * 1053 #	C–O stretching and C–OH bending (nucleic acids, phospholipid phosphate, glycogen, and other carbohydrates)	[26,27,28]
1039 # 1031 *	C–O stretching (lipid, carbohydrate) C–O stretching and C–OH bending (nucleic acids and carbohydrates) Glycogen vibration	[24,26,27,29]
1022 #	C–O stretching and C–OH bending (nucleic acids and carbohydrates) C–O and C–C stretching and C–O–H deformation (glycogen)	[26,27,28]
991 * 988 #	C–O stretching from RNA ribose chain and other carbohydrates (nucleic acids ribose) RNA ribose phosphate main chain mode C–C stretching of DNA backbone	[26,27,28,29]
969	${\mathrm{PO}}_{3}^{2-}$ symmetric stretching of nucleic acids and phosphorylated proteins C–C, C–O, and C–N–C stretching of phosphodiester, deoxyribose and ribose (nucleic acids)	[26,27,29,30]
929 * 923 #	Left-handed helix DNA (Z-form)	[27]
915 *	Ribose ring vibrations (nucleic acids)	[27,29]

# FTIR peak observed in N2a; * FTIR peak observed in N2a-derived EVs.

**Table 2:** Parameters of PLS classification models of factor 1 for N2a and N2a-derived EVs for 1280–900 cm^−1^ spectroscopic region.

	Correlation		RMSEC/RMSECV	
	Calibration	Validation	Calibration	Validation
N2a	0.667	0.523	0.373	0.437
EVs	0.670	0.582	0.371	0.412

RMSEC, Root Mean Squares Error of Calibration; RMSECV, Root Mean Squares Error of Cross-Validation.

## Data Availability

The original contributions presented in the study are included in the article, further inquiries can be directed to the corresponding author.

## Supplementary Materials

The following supporting information can be downloaded at: https://www.mdpi.com/article/10.3390/molecules30020258/s1, Figure S1: N2a cells viability upon Aβ treatment.
