# Supplementary material for: Fourier Transform Infrared Spectroscopy Analysis as a Tool to Address Aβ Impact on Extracellular Vesicles
Source: Molecules. 2025 Jan 10;30(2):258. doi: 10.3390/molecules30020258 (PMC11767854; doi:10.3390/molecules30020258)
Supplement: Supplementary file 1 [file molecules-30-00258-s001.zip › molecules-3325076-supplementary.pdf]

molecules

Article

FTIR Spectroscopy Analysis as a Tool to Address Aβ Impact on Extracellular Vesicles

Margarida Vaz, Tânia Soares Martins, Kevin Leandro, Luís Pereira de Almeida, Odete da Cruz e Silva, Alexandra Nunes and Ana Gabriela Henriques

Supplementary Material

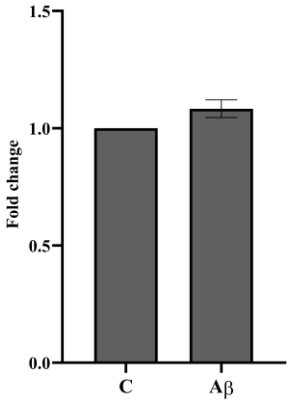

Figure S1. N2a cells viability upon Aβ treatment. Cell viability was measured using resazurin assay by incubating cells for 4 h (following Aβ treatment) with fresh medium containing 10% of a resazurin solution [0.1 mg/mL resazurin (Sigma-Aldrich, St. Louis, MO, USA) in PBS]. Resazurin conversion to resorufin was calculated by measuring conditioned medium absorbance at 570 and 600 nm using Infinite M200 PRO (Tecan, Männedorf, Switzerland) and calculating the ratio O.D. 570/O.D. 600 nm. The quantity of resorufin produced is proportional to the number of viable cells. Fold change to control is expressed as mean ± standard deviation of 3 independent experiments.

 https://doi.org/10.3390/molecules30020258
